# Can Sex Be Determined from a Blood Smear?

**DOI:** 10.4274/tjh.2011.0015

**Published:** 2013-03-05

**Authors:** Mohamed Brahimi, Affaf Adda, Hassiba Lazreg, Hadjer Beliali, Soufi Osmani, Mohamed Amine Bekadja

**Affiliations:** 1 EHU "1er Novembre 1954" - Hemobiology, Oran, Algeria; 2 EHU "1er Novembre 1954" - Hematology and Cell Therapy, Oran, Algeria

**Keywords:** gender, Nuclear appendages, Polymorphonuclear neutrophils, Blood Smear, May-Grünwald-Giemsa stain

## Abstract

**Objective: **Originally, this blind study was designed to check whether blood smears constitute reliable tools to determine sex. However, when we analyzed our data some interesting findings immerged and in this paper we try to highlight them.

**Material and Methods:** 74 blood smears (35 women and 39 men) have been performed and then stained. 200 polynuclearneutrophils were examined for nuclear appendages and classified into four groups: neutrophils with form A, B or C appendages and neutrophils without any appendage.The difference (A-C) was calculated for each slide. The “cytologic sex” was defined as a male in case of a negative value and as a female otherwise.

**Results:** Neutrophils bear the same amount of appendages in both genders (p=0.37). But the number of form A is greater in females (p<0.0001) and form C is much more frequent in males (p<0.0001), that is why, the difference A-C is the best way to differentiate between both sexes.The distribution histogram of A-C in women shows a multimodal histogram contrary to men’s graphwhich is a bell-shaped curve. The menstrual cycle was incriminated in this feature.

**Conclusion:** Blood smear is a reliable tool to determine gender.

**Conflict of interest:**None declared.

## Abstract (tr)

**Amaç:** Orijinal olarak planlanmış bu kör çalışmanın amacı çevre kan yayması incelemesi ile cinsiyet belirlemesinin yapılıp yapılamayacağını tespit etmek. Elimizdeki bulguları incelediğimizde bazı ilginç sonuçlar ortaya çıkmıştır; bu çalışmada onları vurgulamak istiyoruz.

**Gereç ve Yöntemler:** 74 çevre kan yayması (35 kadın; 39 erkek) hazırlandı. 200 nötrofil çekirdek ilave uzantısı açısından incelendi ve dört gruba ayrıldı: A, B, C tarzında çekirdek ilave uzantısı olanlar ve hiç çekirdek ilave uzantısı olmayanlar. Her çevre kan yaymasındaki çekirdek ilave uzantısı şekillerindeki (A-C) tarzındaki farklılıklar sayısal olarak tespit edildi. Eğer hesaplanan değer negatif ise ‘sitolojik cinsiyet’ erkek olarak tanımlandı, diğer durumlarda ise kadın olarak tanımlandı.

**Bulgular:** Nötrofiller her iki cinsiyette de aynı miktarda çekirdek ilave uzantısı taşımaktaydı (p=0,37). Fakat A şekli kadınlarda (p<0,0001); C sekli ise erkeklerde daha sık gözlendi (p<0,0001). O yüzden A-C histogramının dağılımı kadınlarda multimodal histogram seklindeyken, erkeklerdeki dağılım grafiği çan-eğrisi seklindeydi. Bu farklılıktan menstrüel durum sorumlu tutuldu.

**Sonuç:** Çevre kan yayması incelemesi cinsiyet tayininde güvenilir bir yöntemdir.

## INTRODUCTION

Each time granulocytes are described in a hematology atlas book, nuclear appendages are mentioned [1,2]. Some polymorphonuclear neutrophils contain a small chromatin mass (1.5 µm) appended to one of their nucleus lobes. To date, their nature has remained uncertain. According to some authors, these appendages are assumed to be constituted of sex chromatin derived from heterochromatin proportions of the XX chromosome complex [3,4].

However, some published data demonstrated that the frequencies and the distribution of these appendages were not influenced by sex only, but also by many other factors such as hormones, granulocytes metabolism, cell proliferation, and age [5].

Originally, this blind study was designed to check whether blood smears constitute reliable tools to determine sex. However, when we analyzed our data some interesting findings emerged, and in this paper, we try to highlight them. 

## MATERIALS AND METHODS

**Blood Samples and Preparation of Smears**

Seventy-four blood samples, from 35 women and 39 men, were haphazardly selected from the thousands of tubes submitted to us for complete blood counts. All of these samples belonged to patients hospitalized in our establishment regardless of their illness or their treatment. The ages of the studied patients varied from 16 to 80 years old. 

All venous blood specimens were collected into tubes containing ethylenediaminetetraacetic acid (K2 or K3EDTA). Thin air-dried blood smears were made, labeled only by their tube number, and then stained manually with May-Grünwald-Giemsa stain.

**Slide Examination**

The smears were examined under light microscopy by one of the authors in a blinded manner in order to determine “cytologic sex” for each sample. The slides were entirely scanned with 100× oil-immersion lens for abnormal nucleated cells such as blast cells, normoblasts, or immature granulocytes and, if any were found, the samples were excluded from the study.

**Figure 1:**
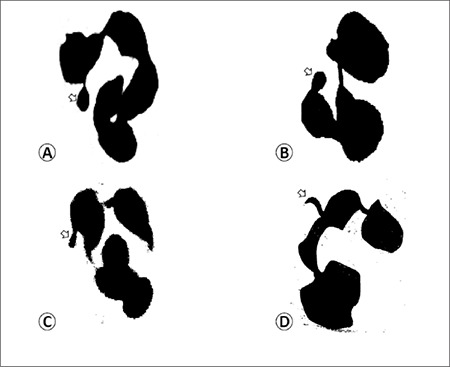
Different forms of appendages (arrow): A) form A (drumstick), B) form B (sessile nodules), C) and D) form C (C: tag and D: hook).

A total of 200 polynuclear neutrophils were examined for nuclear appendages and classified into 4 groups: neutrophils with form A, B, or C appendages and neutrophils without any appendages. Figure 1 shows the different forms of appendages.

The difference of A–C was calculated for each slide. The “cytologic sex” was defined as male in the case of a negative value and as female otherwise.

**Statistical Methods **

For statistical evaluation, an online calculator and free graphing software were used [6]. 

Student’s t-test was performed in order to assess the difference between data for both sexes. Error boxes (mean±2 standard deviation error bars) with data swarm were plotted in order to compare men’s and women’s data. A difference (A – C) distribution histogram was used to compare the distribution curves in both sexes. 

## RESULTS

The Student’s t-test results are represented in Table 1, which shows that neutrophils bear the same amount of appendages in both sexes. However, the number of form A appendages is greater in females (p<0.0001) and form C is much more frequent in males (p<0.0001); this is why the difference of A – C is the best way to differentiate between the sexes.

**Figure 2:**
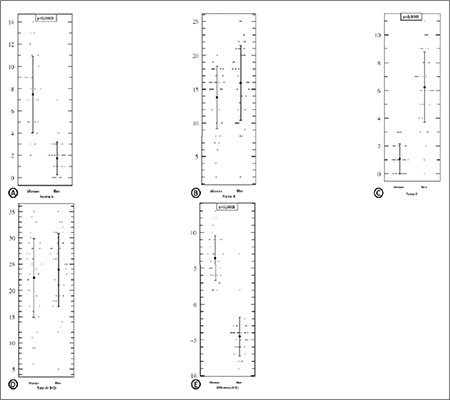
Error boxes (means ± 2 standard deviation error bars) with data swarm. Each dot represents a subject. The left bar represents the women’s data and the right the men’s data. Error bars are centered on the mean of the distribution range and demonstrate an interval of 2 standard deviations of the mean.

Figures 2A-2C show error boxes with data swarm of a single parameter. They show that form A is more frequent in women than in men, whereas form C is much more frequent in men and there is no difference in form B frequencies in the sexes.

The error box of the sum (A + B + C) shows that there is no significant difference in the proportion of neutrophils bearing appendages (Figure 2D).

Figure 2E shows that the difference of A – C is the best way to differentiate between the sexes, whereby A – C gives a positive value in all females and a negative value in all males except for 2. The difference (A – C) was equal to +2 in 1 man and +7 in another. These later were considered as odd cases.

**Figure 3:**
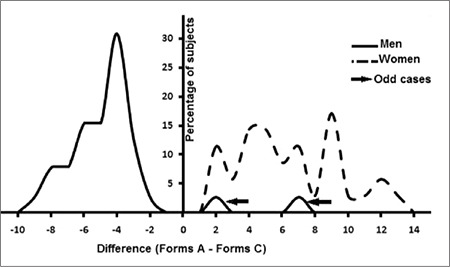
Distribution histogram of the difference of A – C. The solid curve represents men’s data and the dashed curve represents women’s data.

Figure 3 represents percentages of subjects versus A – C value curves in both sexes. The men’s histogram looks like a bell-shaped curve whereas the women’s is a multimodal curve. 

Table 1 and Figure 2E show that the A – C difference is the best way to differentiate between the sexes (t=16.1 and M* – M§ = 10.94), comparing counts of forms A and C. 

## DISCUSSION

Davidson and Smith were the first to identify the peculiar nucleus projections in neutrophils and define their relationship with sex chromatin of the cells [7]. The nomenclature used in this study is derived from Kosenow’s formula [8]. Thus, drumsticks are called “form A”: these are stalked, round-headed appendages of chromatin, 1.5 µm in diameter, attached to a nucleus lobe with a thin stem (Figure 1A) [9]. Sessile nodules, or “form B”, were described by Davidson and Smith as having the appearance of drumsticks but being devoid of any stem (Figure 1B) [9]. Leukocytes with other pedunculated nuclear projections, which are easily distinguished from small lobes and forms A and B, such as clubs and hooks, are designated as “form C” (Figures 1C and 1D) [10].

In our study, the leukocytes of both sexes bore nearly the same frequency of appendages (Table 1), but t-tests showed that numbers of form A were greater in females than in males. These results are in agreement with those reported in the literature and confirm the fact that drumstick count is certainly related to sex [7,9]. 

Briggs declared that drumsticks are never seen in male leukocytes [9], but this notion is in contradiction with our findings and those of many other investigators. In our series, in accordance with the findings of others, 30 men out of 39 had drumsticks in 0.5% to 2% of their polymorphs [5,11]. Tomonaga et al. examined 50 men’s blood smears; they found that the frequency of form A varied from 0 to 6 per 1000 polynuclear neutrophils [11]. Gonzalez et al. studied 38 blood smears of newborns (19 males and 19 females) and declared that a great number of “small drumsticks” was found in smears from newborns that were subsequently identified as males [5]. This might be due to the fact that the form A seen in male leukocytes are not “true drumsticks”. Mukherjee and San Sebastian named these male form A projections “drumstick-like” [12]. That is because their structures are similar to typical drumsticks in shape but generally smaller in size. 

Confirmation of the inactive X chromosome in the drumstick and the Y chromosome in the drumstick-like chromosome has been provided by fluorescence in situ hybridization [2,13,14]. Females with an isochromosome of the long arms of the X chromosome have larger and more frequent drumsticks, whereas females with deletions from the X chromosome have smaller drumsticks [1,15]. 

Wondergem and Ossenkoppele reported, in March of 2011,the case of a woman with a myelodysplastic syndrome–myeloproliferative disorder. The presence of double drumsticks(2 drumsticks in 1 cell) prompted a cytogenetic study that showed 47, XXX anomaly [16].

As one can see from our data, the incidence of A appendages varies widely from one woman to another (from 2 to 14 drumsticks per 200 neutrophils) (Figure 2A). These variations had been reported under different circumstances: during the menstrual cycle, after the administration of adrenocorticotropic hormone and insulin, with senility, and with cachexia [5]. 

The majority of investigators believe that “sessile nodules” are as equally sex-specific as drumsticks, but they are more difficult to recognize since they might mingle with the nucleus during a manual spreading of the blood film [1,2,9]. Because of this, only form A is considered for sex diagnosis.

In this study, the t-test did not show any significant difference in the frequency of form B between men and women. This might be due to the fact that some drumsticks, hooks, and tags could be partially hidden underneath the nucleus membrane, which would let them look like sessile nodules.

Many authors consider that only drumsticks are related to the sex chromatin [2,7,9], but if we analyze the series of Tomonaga et al., it can be seen that form C is more frequent in male smears than in female, which is in agreement with our data (Table 1, Figure 2C) [11]. Student’s t-test shows that form C is of equal sex diagnostic significance as form A (Table 1).

It had been reported that an increase in the incidence of small clubs and hooks (form C) could be observed after intensive androgen treatment in humans and animals [17,18]. Méhes studied the blood smears of female patients receiving chronic androgen therapy for mammary carcinoma and compared them with those of untreated controls [10]. The incidence of form C proved to be higher in the androgen-treated patients. He also described a blood smear of a woman with an androgen-producing tumor of the adrenal gland, where 24% of the neutrophils had 1 or more “C” nuclear projections. After the removal of the tumor, the incidence of form C decreased to 8% [10]. These observations prove that the increase in the number of nuclear C appendages might be a manifestation of a high androgen level in men, too.

All of these arguments suggest that the difference in A – C gives a positive value in females and a negative value in males. All subjects followed this rule, except for 2 men who were erroneously classified as females by cytology. One of them had no form C appendages and 7 form A appendages, and the other had 1 form C and 3 form A. These were designated as odd cases.

As was mentioned above, appendage counts fluctuate during menstrual cycle for form A and with androgen level for form C. The distribution histogram of A – C in women shows a multimodal histogram contrary to the men’s graph, which is a bell-shaped curve (Figure 3). This might be due to the overlapping of several groups of females during different phases of the menstrual cycle, whereas men constitute a single population. This notion must be confirmed with further investigations, such as repeating and comparing blood smears of some females in different phases of the menstrual cycle. 

We would like to end our discussion with this mysterious and unanswered question: Why do some few neutrophils bear appendages while others do not?

**Conflict of Interest Statement**

The authors of this paper have no conflicts of interest, including specific financial interests, relationships, and/ or affiliations relevant to the subject matter or materials included.

## Figures and Tables

**Table 1:**
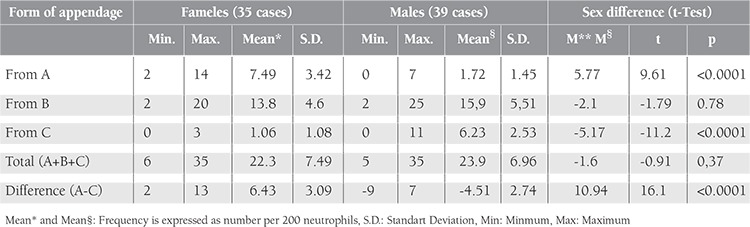
Frequency of each nuclear appendage of neutrophils and sex difference.
